# Construction and validation of a metabolic-related genes prognostic model for oral squamous cell carcinoma based on bioinformatics

**DOI:** 10.1186/s12920-022-01417-3

**Published:** 2022-12-24

**Authors:** Jingfei Zhang, Chenxi Ma, Han Qin, Zhi Wang, Chao Zhu, Xiujuan Liu, Xiuyan Hao, Jinghua Liu, Ling Li, Zhen Cai

**Affiliations:** 1 Department of Stomatology, Binzhou Medical University, Yantai, 264000 Shandong China; 2 Department of Stomatology, Linyi People’s Hospital, Linyi, 276000 Shandong China; 3 Department of Human Microbiome, School and Hospital of Stomatology, Shandong Provincial Key Laboratory of Oral Tissue Regeneration, Shandong Engineering Laboratory for Dental Materials and Oral Tissue Regeneration, Shandong University, Jinan, 250000 Shandong China; 4 Department of Hepatobiliary Surgery and Minimally Invasive Institute of Digestive Surgery and Prof. Cai’s Laboratory, Linyi People’s Hospital, Shandong University, Linyi, 264000 Shandong China

**Keywords:** Metabolic studies, Oral squamous cell carcinoma, Prognosis, Nomogram, Bioinformatics

## Abstract

**Background:**

Oral squamous cell carcinoma (OSCC) accounts for a frequently-occurring head and neck cancer, which is characterized by high rates of morbidity and mortality. Metabolism-related genes (MRGs) show close association with OSCC development, metastasis and progression, so we constructed an MRGs-based OSCC prognosis model for evaluating OSCC prognostic outcome.

**Methods:**

This work obtained gene expression profile as well as the relevant clinical information from the The Cancer Genome Atlas (TCGA) database, determined the MRGs related to OSCC by difference analysis, screened the prognosis-related MRGs by performing univariate Cox analysis, and used such identified MRGs for constructing the OSCC prognosis prediction model through Lasso-Cox regression. Besides, we validated the model with the GSE41613 dataset based on Gene Expression Omnibus (GEO) database.

**Results:**

The present work screened 317 differentially expressed MRGs from the database, identified 12 OSCC prognostic MRGs through univariate Cox regression, and then established a clinical prognostic model composed of 11 MRGs by Lasso-Cox analysis. Based on the optimal risk score threshold, cases were classified as low- or high-risk group. As suggested by Kaplan–Meier (KM) analysis, survival rate was obviously different between the two groups in the TCGA training set (*P* < 0.001). According to subsequent univariate and multivariate Cox regression, risk score served as the factor to predict prognosis relative to additional clinical features (*P* < 0.001). Besides, area under ROC curve (AUC) values for patient survival at 1, 3 and 5 years were determined as 0.63, 0.70, and 0.76, separately, indicating that the prognostic model has good predictive accuracy. Then, we validated this clinical prognostic model using GSE41613. To enhance our model prediction accuracy, age, gender, risk score together with TNM stage were incorporated in a nomogram. As indicated by results of ROC curve and calibration curve analyses, the as-constructed nomogram had enhanced prediction accuracy compared with clinicopathological features alone, besides, combining clinicopathological characteristics with risk score contributed to predicting patient prognosis and guiding clinical decision-making.

**Conclusion:**

In this study, 11 MRGs prognostic models based on TCGA database showed superior predictive performance and had a certain clinical application prospect in guiding individualized.

## Background

Oral cancer (OC) accounts for the head and neck cancer that mainly originates from the cheek, tongue, lip and palate. There are more than 300,000 new oral cancer cases and more than 145,000 related deaths annually [1]. Oral squamous cell carcinoma (OSCC) occupies ninety percent of OC patients. The traditional treatment of OSCC is mainly surgical resection of the tumor, preoperative or postoperative chemotherapy, radiotherapy and adjuvant therapy [2]. As researchers continue to study this disease, the treatment options for these patients continue to improve. Image-based and adaptive radiotherapy, transoral robotic resection and immunotherapy are gradually being utilized to treat OSCC [3–5]. Unfortunately, most patients are diagnosed at a late stage, the local recurrence rate is high, and metastasis often occurs, making the 5-year survival rate much lower than that of other malignant tumors. In fact, during the previous 30 years, the survival rate of oral squamous cell carcinoma patients has been consistently less than 50% [6, 7]. More accurate prediction of the prognosis of OSCC patients will allow doctors to better choose appropriate treatment strategies and improve the survival rate of patients [8]. Currently, the tumor, node, metastasis (TNM) classification system has been mainly used to predict tumor prognosis and provide clinical guidance in choosing appropriate treatment methods. However, the OSCC-related TNM system mainly focuses on the anatomical extent of the disease and ignores factors related to tumor prognosis, such as age, gender, and the presence of other diseases. Thus, patients who have the same TNM stage can have very different survival outcomes; in other words, the use of the TNM staging alone for the prediction of patient survival is insufficient [9]. The eighth American Joint Commission on Cancer (AJCC) staging system emphasizes the need for a "personalized" treatment approach for cancer patients [10]. In addition, inaccurate prognostic information can affect treatment decisions and subsequent outcomes. For example, high-risk cases are possibly associated with cancer migration and metastasis due to insufficient or delayed treatment, and low-risk patients may experience a loss of bone marrow function and organ function because of excessive treatment; both situations have substantial effect on patient treatment or recovery [11]. Consequently, it is urgently needed to construct the creditable prognostic approaches that can assist clinicians in selecting the suitable individualized therapeutic strategies, thus improving OSCC prognosis.

Changes in metabolic processes are closely related to tumor growth [12–15]. During the process of rapid growth and proliferation of tumor cells, the metabolic pathways of the body are in constant flux. In other words, during the process of tumorigenesis, progression and metastasis, tumor cells reprogram catabolic and anabolic pathways to satisfy the energy metabolism and biosynthesis of cells by enhancing macromolecular biosynthesis, regulating the redox balance and allowing for the rapid production of ATP [16, 17]. In the 1920s, Otto Warburg discovered that under both aerobic and anaerobic conditions, the ATP produced by tumor cells tends to undergo glycolysis rather than oxidative phosphorylation, which offers more energy for the rapid proliferation of tumor cells; this has been termed the “Warburg effect” [18]. In addition, the metabolic processes involved in tumor metabolic reprogramming include the pentose phosphate pathway, lipid metabolism, and nucleic acid and amino acid metabolism [19]. During tumorigenesis, progression and metastasis, changes in MRGs determine the changes in metabolic pathways [20]. For example, some studies have shown that *PER1* and *PER2* are significantly downregulated in OSCC and that their overexpression can inhibit glycolysis and tumor cell proliferation, thus inhibiting tumor growth [21, 22]. In contrast to *PER1* and *PER2*, the expression of *SHMT2* in OSCC was found to be significantly upregulated, which predicts the dismal OSCC survival [23]. In addition, the upregulation of *PFKP* in OSCC is related to the pathological differentiation of tumors as well as lymph node metastasis [24]. According to the obtained results, metabolism-related genes (MRGs) are a prognostic marker and therapeutic target for OSCC.

As bioinformatics analysis is increasingly used for diagnosing and predicting cancer prognostic outcome, some researchers have connected metabolome with genome [25]. To date, many studies have used MRGs to construct clinical prognostic models of malignant tumors like gastric cancer, cervical cancer, liver cancer and renal clear cell carcinoma, and these studies have achieved good predictive results [26–28]. However, the development of clinical prognostic models for OSCC based on MRGs has been barely explored and are not comprehensive [29]. In this study, clinical data and gene levels for OSCC cases were acquired in The Cancer Genome Atlas (TCGA). This TCGA-OSCC cohort was then used as the training set to screen the differentially expressed MRGs that were closely related to OSCC by difference analysis. The MRGs found to be significantly related to prognosis were screened by univariate Cox analysis, and the clinical prognosis model of OSCC based on 11 MRGs was constructed by Lasso-Cox analysis. Finally, risk score was calculated and identified as the factor that independently predicted OSCC prognosis upon univariate as well as multivariate Cox regression. In addition, we further validated the dataset obtained using the Gene Expression Omnibus (GEO) database as the validation set. This study describes a new approach to predict overall survival (OS) of OSCC cases, which could help direct the individualized treatment of OSCC patients.

## Materials and methods

### Data downloading and processing

This work obtained clinical data together with the FPKM-normalized gene profiles for 340 cancers along with 32 non-carcinoma samples in TCGA database OSCC cohort (https://portal.gdc.cancer.gov/). Apart from that, this work also obtained the GSE41613 dataset, which includes 97 OSCC samples, in GEO database (https://www.ncbi.nlm.nih.gov/geo/). According to annotation patterns obtained by relevant platform, this work transformed the gene matrix file of probe identification as gene symbols. In addition, samples collected in cases with a < 90-day follow-up period from TCGA-OSCC cohort and GSE41613 were excluded, and the survival time was changed from years/months to days. The TCGA-OSCC cohort was enrolled into training cohort, while the GSE41613 dataset into external validation cohort. This work acquired a total of 851 MRGs (c2.cp.kegg.v7.4.symbols.gmt) in GSEA (http://www.gsea-msigdb.org/gsea/index.jsp).

### Screening differentially expressed genes (DEGs)

After genes from training cohort were intersected with those from metabolic gene cohort, the MRGs in the TCGA gene expression profile were extracted. This work employed R software (Version 4.1.2) limma R package for screening metabolic DEGs within cancer compared with non-carcinoma samples upon |log_2_FC|> 0.5 and FDR < 0.05 thresholds. Then, we used the glmnet R package to integrate survival status, survival time, as well as DEGs expression profiles.

### Construction of the MRGs-based prognostic signature

By univariate Cox regression, this work calculated hazard ratios (HRs) as well as relevant 95% confidence intervals (CIs) and obtained 12 genes related to overall survival. Next, we selected the most appropriate lambda value to obtain the optimal model through lasso analysis, and screened the above 12 genes into 11 MRGs. Meanwhile, the present study calculated risk score by the gene correlation coefficient obtained by Lasso-Cox analysis. Survminer R and survival R packages were utilized to conduct Kaplan–Meier (KM) analysis, and the cutoff value of continuous variables in survival data was measured through survminer R package surv_cutpoint function. In addition, pROC package roc function was also adopted for analyzing 1, 3 and 5 years receiver operating characteristic (ROC) curves, separately, whereas area under the curve (AUC) values and CIs were adopted for calculating pROC package ci function for obtaining the final AUC results and determining risk score’s sensitivity and accuracy in the prediction of OSCC prognosis at 1, 3 and 5 years. Then, this work carried out univariate as well as multivariate Cox analysis for comparing the association of risk score with additional clinicopathological factors in prognosis prediction for training set (including age, gender, stage, grade, T stage and N stage). Finally, association of risk score based on MRGs with clinical factors was analyzed for better confirming that our prognosis model was accurate. M stage was excluded because of the lack of data.

Taking GSE41613 as the verification set, this work determined risk score by the calculation formula used for training cohort. All cases were classified as low- and high-risk groups based on optimal threshold. ROC survival analysis was carried out on the verification set.

### Construction and verification of nomogram

According to age, gender, risk score and TNM stage, R software rms package was adopted to integrate the data of seven characteristics, such as survival status or survival time. This work then predicted 1, 3 and 5 year survival for OSCC cases by adding the total scores of the points of each factor into that as-constructed nomogram. The high score indicates the low survival probability. Thereafter, we evaluated the nomogram performance in prognosis prediction by ROC curves and calibration curves.

### Enrichment analysis

With the purpose of understanding the gene function in the model, R software was employed to conduct enrichment analysis by adopting Gene Ontology (GO) along with Kyoto Gene and Genomic Encyclopedia (KEGG) databases to obtain the gene set enrichment results. Then, we used GSEA (Version 4.1.0) software for the analysis. *P* < 0.05 was defined as a significantly enriched pathway to explore the differences in the low-risk and high-risk groups in the training set.

### Immune cell infiltration

Single-sample gene set enrichment analysis (ssGSEA) was used to calculate the relative ratio of infiltrating immune cells.

### Single-cell sequencing and cell type identification

ScRNA-Seq data from the GSE172577 was obtained from the GEO database. Seurat package is used to process the expression matrix of. Cells that meet the following conditions (> 15,000 UMI/cell, < 6000 genes/cell, > 6000 genes/cell and > 20% mitochondrial genes) were considered low-quality cells and were removed. DoubletFinder package was used to identify and remove potential doublets. We use principal component analysis (PCA) to reduce the dimensionality of the scRNA-Seq dataset. Top 30 principal components (PCs) were used for UMAP. The main cell clusters were identified with the FindClusters function offered by Seurat.

### Statistical analysis

R software (Version 4.1.2) was employed for performing statistical analysis. KM curve analysis and log-rank test were performed for analyzing different survival rates between both groups. p-value was determined according to two-sided statistical tests. Except for the statistical standards specified, all statistically significant results needed to satisfy *P* < 0.05.

## Results

### Identification of differentially expressed MRGs

**Fig. 1:**
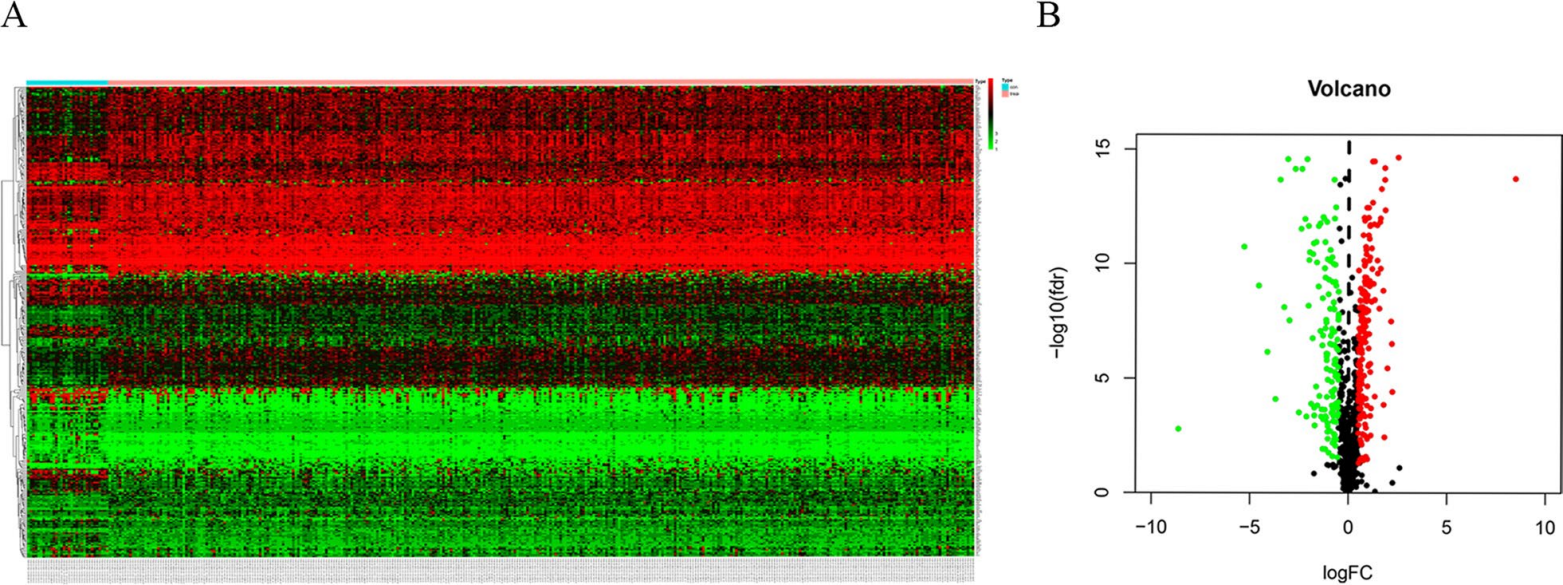
A total of 175 upregulated and 142 downregulated metabolic genes were identified in the tumor dataset compared to the normal dataset, using | log_2_FC |> 0.5, FDR < 0.05 as the screening criteria. **A** Heatmap. **B** Volcano plot

In this study, the training set included 319 patients diagnosed with OSCC, and the GSE41613 validation set included 94 patients diagnosed with OSCC. This work enrolled altogether 413 OSCC cases in both datasets. In the Limma software package, 317 MRGs heatmaps (Fig. 1a) and volcano maps (Fig. 1b) were screened by difference analysis, including 175 up-regulated genes and 142 down-regulated genes.

### Construction and verification of the MRGs-based prognostic signature

**Fig. 2:**
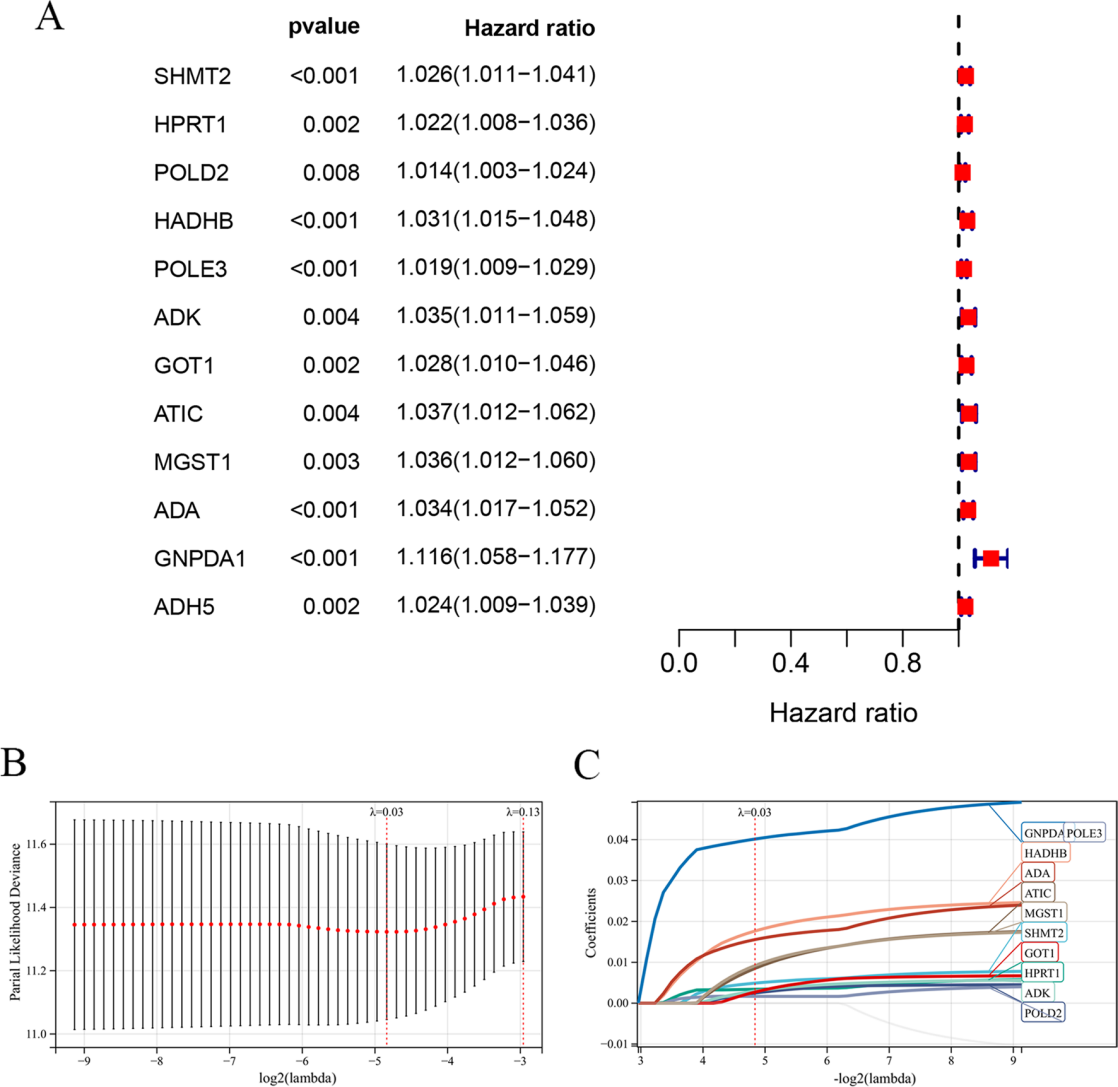
Construction a prognostic signature using univariate Cox regression analysis and Lasso-Cox regression analysis. **A** Risk ratio forest plot shows the prognostic value of 12 candidate genes screened out by univariate Cox regression. **B** Lasso coefficient spectrum of eleven MRGs. **C** On account of 1000 cross-validation for tuning parameter selection via Lasso

**Fig. 3:**
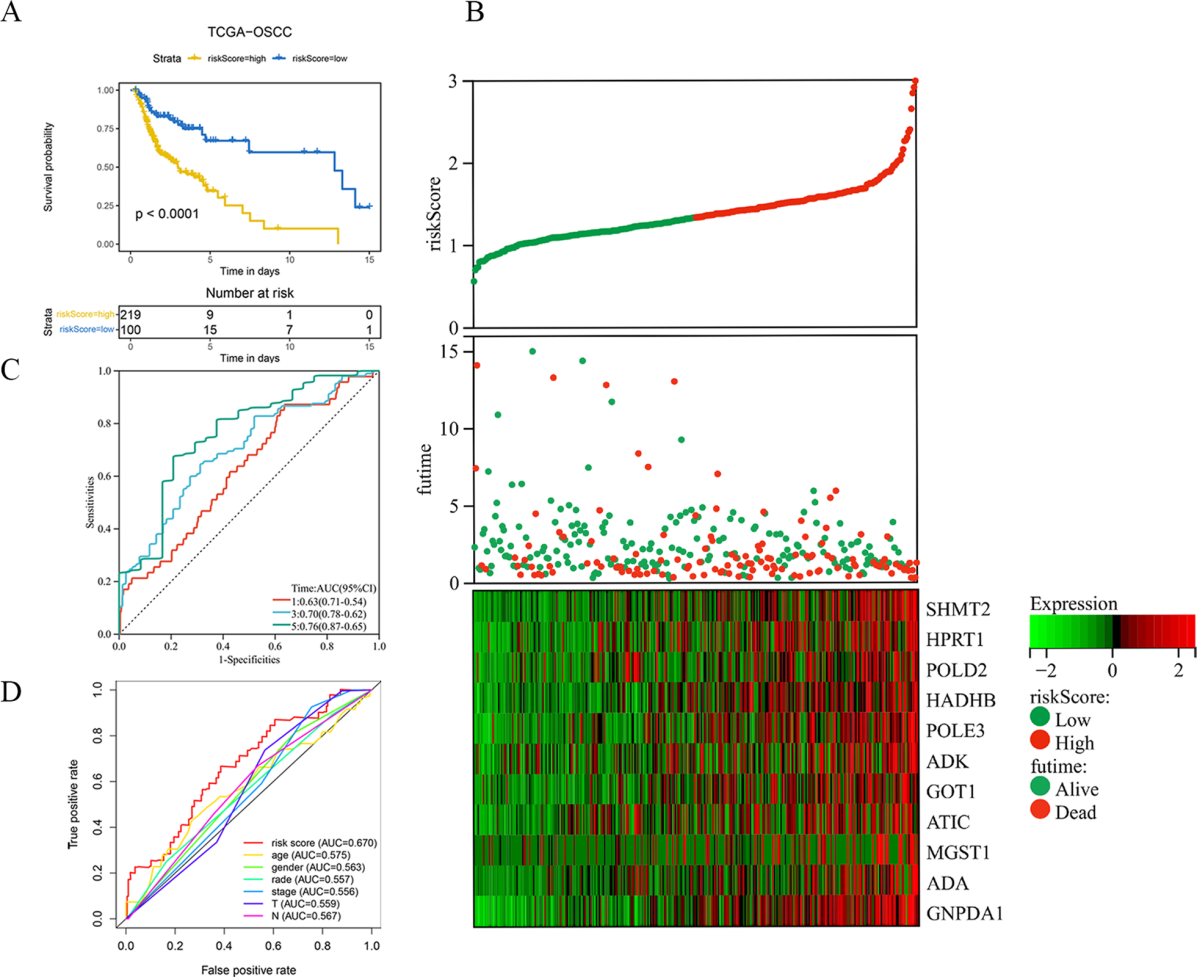
KM survival analysis, risk score assessment by the MRGs risk signature and time- dependent ROC curve in the training set. **A** KM survival analysis of high- and low- risk samples in the training set. Patients in high‐risk group had a shorter OS compared to those in low‐ risk group. **B** Risk scores, survival status distribution, and heat map for prognosis-associated metabolic genes of patients with OSCC in the training set. **C** ROC curve analysis results show the accuracy and reliability of the prognostic signature in determining the 1, 3 and 5 years survival outcomes (AUC values are shown in parentheses). **D** ROC curves of the prognostic signature and clinical risk factors for 1 year survival

The expression of each index within training cohort was examined through univariate Cox regression, as shown in Fig. 2a: Twelve differentially expressed MRGs related to prognosis were detected as potential prognostic molecular markers. Red is used to depict the genes that are positively correlated with a poor prognosis, and the results indicate that all 12 prognosis-related genes screened are risk genes (HR > 1). Eleven prognostic gene models were established by Lasso-Cox analysis, which included *SHMT2*, *HPRT1*, *POLD2*, *HADHB*, *POLE3*, *ADK*, *GOT1*, *ATIC*, *MGST1*, *ADA* and *GNPDA1* (Fig. 2b and c). Table 1 lists the correlation coefficients of diverse genes. Risk scores were determined by using the correlation coefficient of the gene, as follows: (0.004 * expression of *SHMT2* + 0.003 * expression level of *HPRT1* + 0.002 * expression level of *POLD2* + 0.016 * expression level of *HADHB* + 0.002 * expression level of *POLE3* + 0.002 * expression level of *ADK* + 0.001 * expression level of *GOT1* + 0.006 * expression level of *ATIC* + 0.006 * expression level of *MGST1* + 0.014 * expression level of *ADA* + 0.039 * expression level of *GNPDA1*). For training cohort, its risk scores were 0.566–2.989, then cases were classified as low-risk (n = 160) or high-risk (n = 159) group based on the optimal threshold. KM curve analysis in Fig. 3a was conducted for analyzing survival of low- and high-risk patients. As a result, high-risk patients had markedly decreased survival in comparison with low-risk patients (*P* < 0.0001). With regard to risk score, expression pattern and survival status distributions (Fig. 3b), from left to right, the risk scores of the patients were sorted from the lowest to the highest, and the dots represent the OSCC patients. Meanwhile, in heatmap, the green-to-red color stands for low-to-high expression level. Based on the above results, the 11 MRGs showed increased levels with risk score, while OSCC survival declined. As revealed by ROC curves at 1, 3 and 5 years (Fig. 3c), the AUC values of training cohorts were determined to be 0.63, 0.70, and 0.76, separately, which indicated that our as-constructed 11 MRGs-based model served as the favorable prognostic tool with good predictive accuracy and sensitivity. We also constructed a ROC curve (Fig. 3d) involving clinical risk factors like age and gender as well as the risk score. The results showed that compared with age (AUC = 0.575), gender (AUC = 0.563), stage (AUC = 0.556), grade (AUC = 0.557), T stage (AUC = 0.559) and N stage (AUC = 0.567), the risk score had a larger AUC (AUC = 0.670), suggesting that the risk score based on MRGs could have a certain role in predicting prognosis.

**Table 1:** Prognostic associated metabolic genes

	HR	HR.95L	HR.95H	*P* value	Coef
SHMT2	1.026	1.011	1.041	0.001	0.004
HPRT1	1.022	1.008	1.036	0.002	0.003
POLD2	1.014	1.003	1.024	0.009	0.002
HADHB	1.031	1.015	1.048	0.000	0.016
POLE3	1.019	1.009	1.029	0.000	0.002
ADK	1.035	1.011	1.059	0.004	0.002
GOT1	1.028	1.010	1.046	0.002	0.001
ATIC	1.037	1.012	1.062	0.004	0.006
MGST1	1.036	1.012	1.060	0.003	0.006
ADA	1.034	1.017	1.052	0.000	0.014
GNPDA1	1.116	1.058	1.177	0.000	0.039

**Fig. 4:**
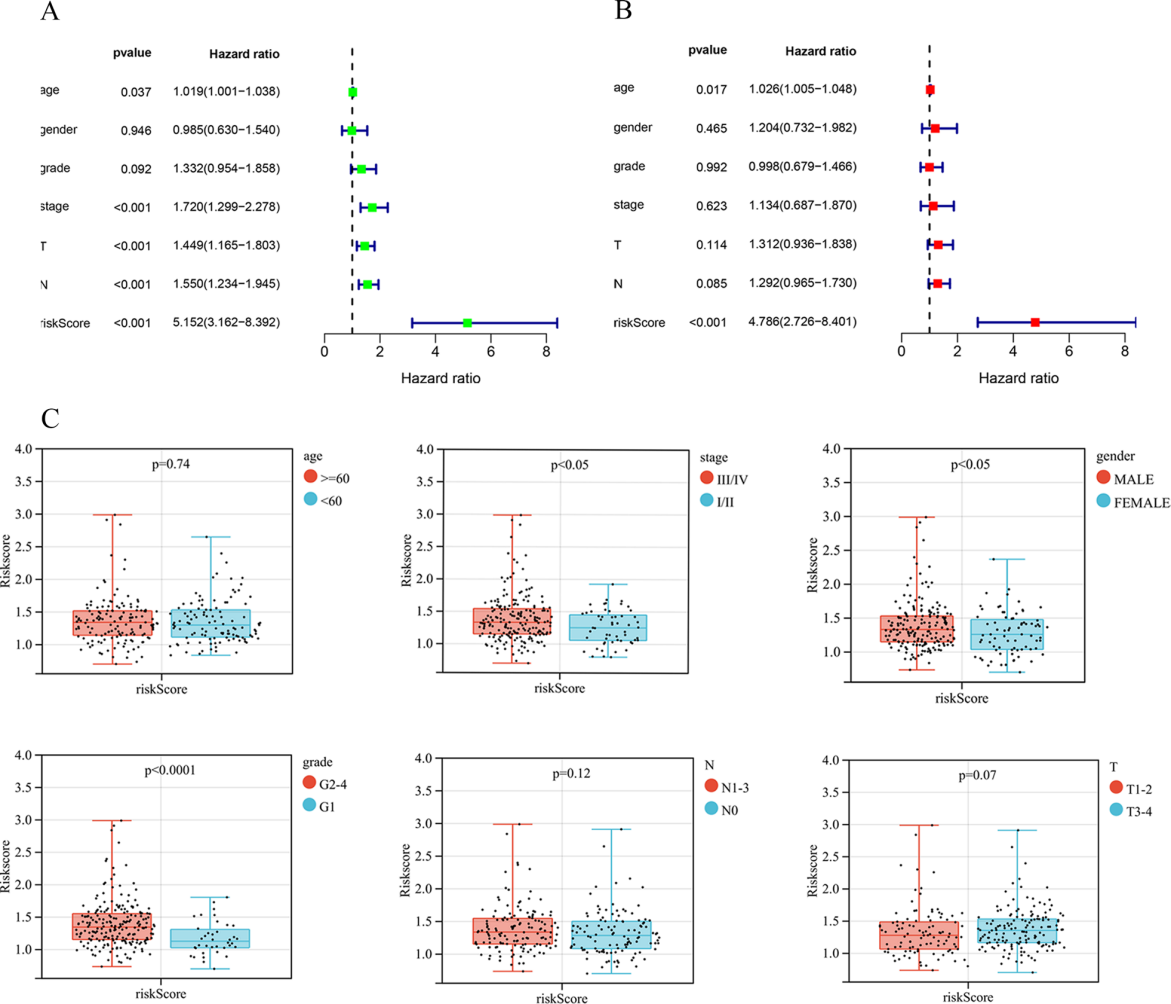
Univariate and multivariate Cox regression analysis of MRGs and clinical significance of the prognostic signature in OSCC patients of training set. **A** univariate Cox regression analysis. **B** multivariate Cox regression analysis. **C** Distribution of the risk score in different clinicopathological features in training set

Samples that had insufficient clinical data and the M stage, and those also had a large amount of missing data (n = 168) were excluded, the clinical information, pathological features and risk scores were analyzed through univariate as well as multivariate Cox regression. As revealed by univariate Cox regression (Fig. 4a), risk score showed significant relation to patient prognosis (HR = 5.125, 95% CI 3.162–8.392, *P* < 0.001). Besides, clinicopathological characteristics like age, grade and stage were adjusted, as a result, risk score remained the factor independently predicting OSCC upon multivariate Cox regression (Fig. 4b) (*P* < 0.001). Clinical correlation analysis further compared the relation of risk score with clinicopathological features like age, gender, stage, and grade. Figure 4c shows that the risk scores based on gender, stage and grade in the training set were statistically significant; the risk score of stage III-IV was obviously higher than that of stage I-II (*P* < 0.05), that of G3-G4 markedly increased compared with G1-G2 (*P* < 0.05), while that of male patients remarkably increased relative to female patients (*P* < 0.05). Therefore, the MRGs-based risk score was tightly associated with clinical features, especially staging, grading and gender.

**Fig. 5:**
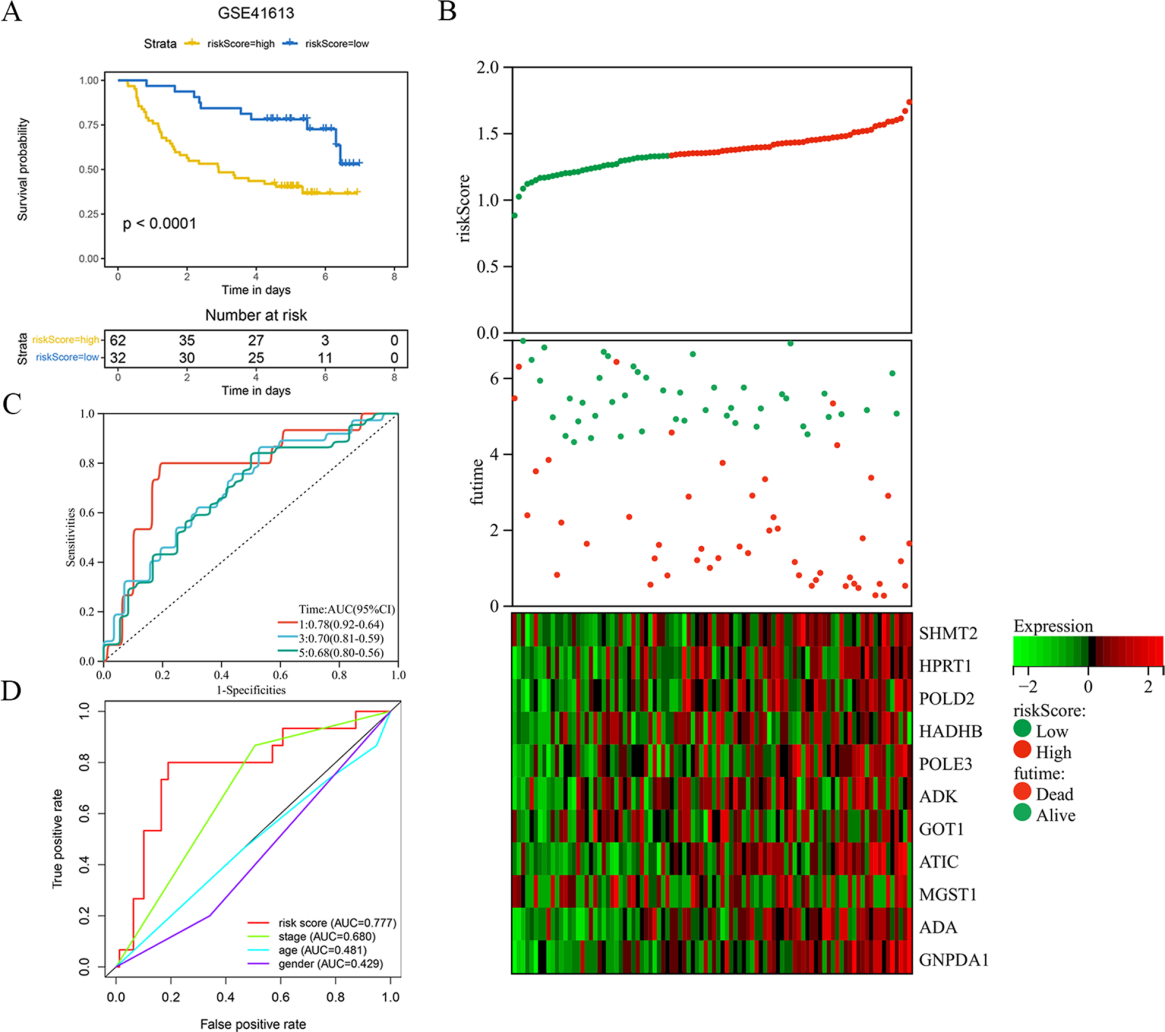
The prognostic signature comprising eleven metabolic genes was verified in the testing set. **A** KM survival curves indicating the overall survival rates of high- and low-risk groups. **B** The ranked dot plot illustrating the predictor‐score distribution, a scatter plot presenting the patients’ overall survival status, a heatmap showing the expression profile of the eleven signature genes of OSCC patients. **C**–**D** ROC curve validation of prognostic value of the prognostic index

**Fig. 6:**
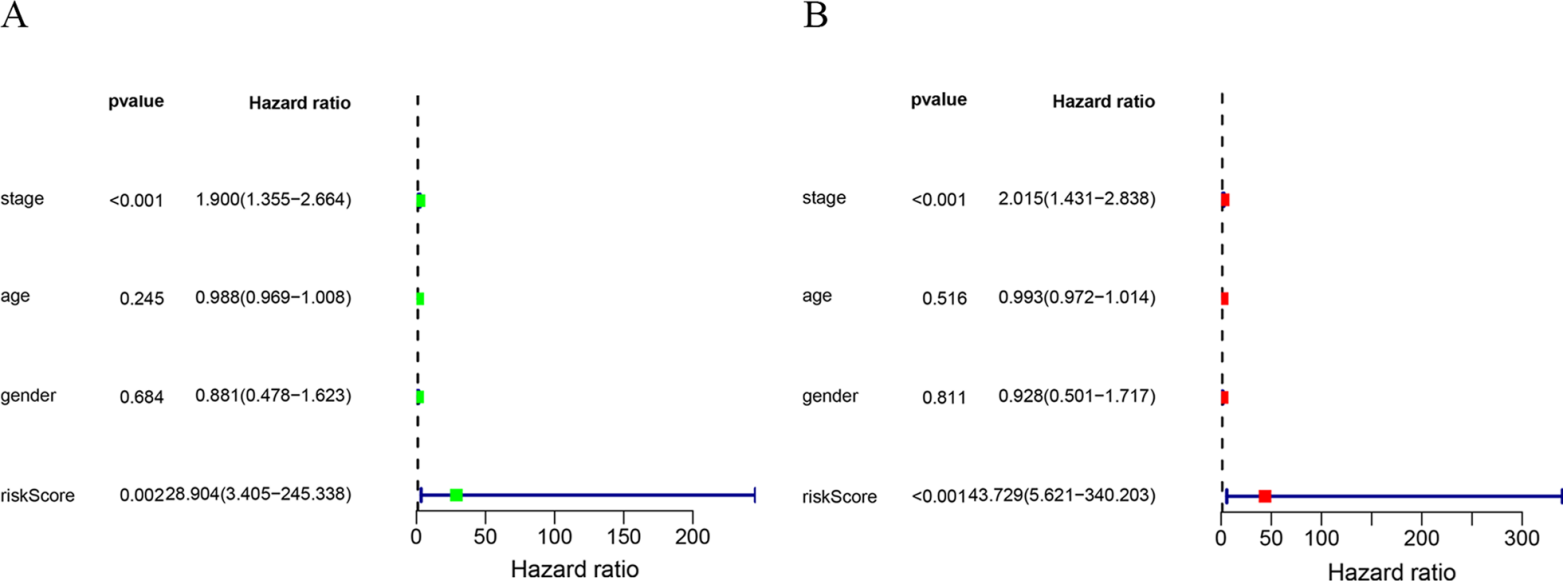
Univariate and multivariate Cox regression analysis of MRGs and clinical indicators based on GEO dataset (GSE41613). **A** Univariate Cox regression analysis. **B** Multivariate Cox regression analysis

For verifying that our as-constructed model was accurate in predicting OSCC prognosis, we used GSE41613 to verify prognosis model. Cases from verification cohorts were classified as low- (n = 37) or high-risk (n = 57) group based on optimal threshold, by adopting identical formula to that in training cohort to calculate risk score. As suggested by the survival curve (Fig. 5a), high-risk cases had decreased survival compared with low-risk cases (*P* < 0.001). Figure 5b presents risk score, survival time and survival status distributions between both groups. As a result, the same trend as the training set was observed; that is, an increased risk score predicted the lower survival rate, and the more frequently the patients expressed risk genes. For ROC curves at 1, 3 and 5 years (Fig. 5c), their AUC values were determined to be 0.78, 0.70, and 0.68, separately, while that for ROC curve of risk score (Fig. 5d) was 0.777, indicating that our model better predicted OSCC prognosis than that based on clinical factors alone. Upon univariate as well as multivariate Cox regression (Fig. 6a and b), risk score was significantly correlated with prognosis. The above results are basically consistent with the verification set, which more full (*P* < 0.001) proves that our model was accurate in prediction.

### Construction and verification of nomogram

**Fig. 7:**
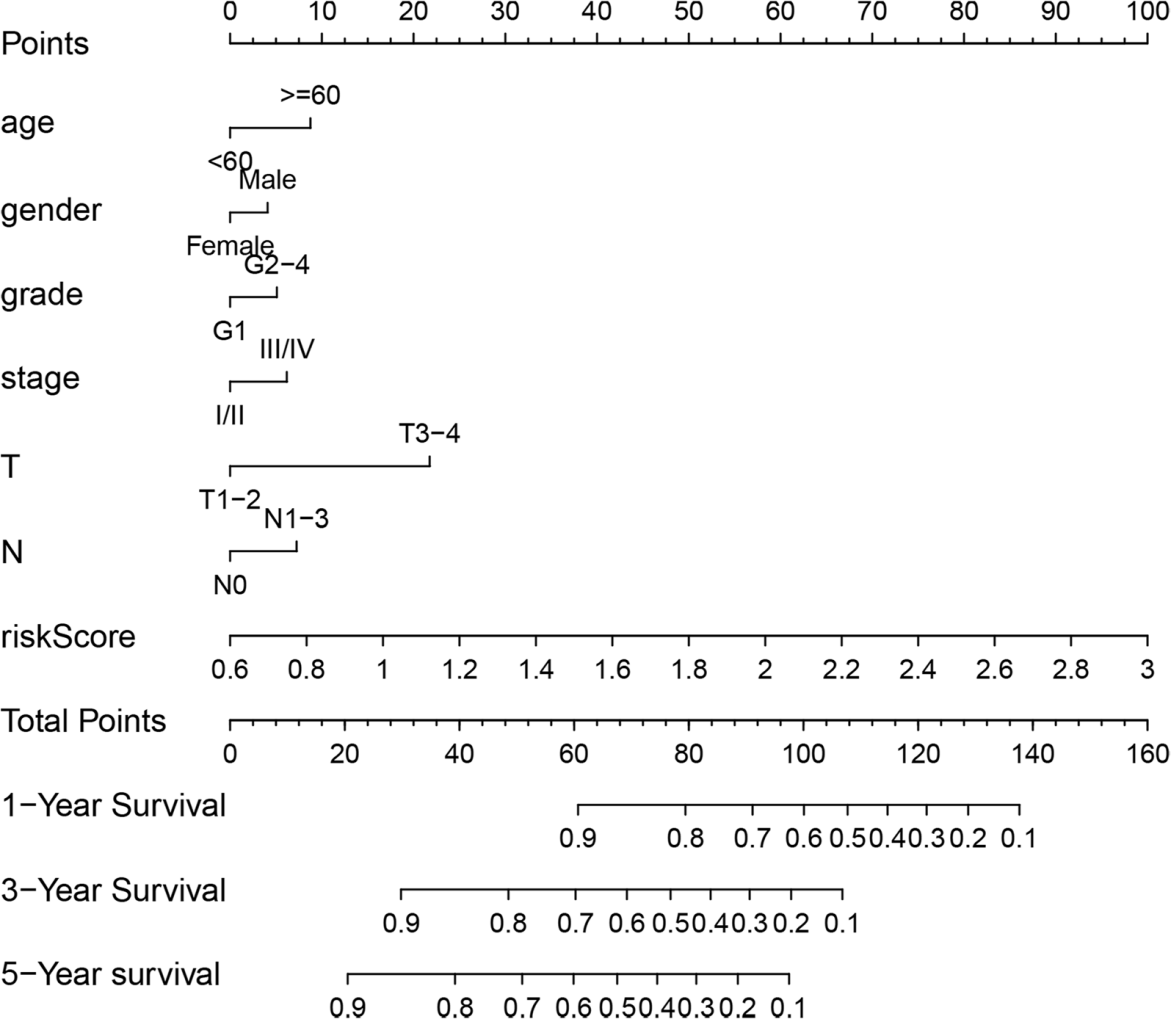
Nomogram for predicting the survival of patients with OSCC. Clinical features (T, grade, and age) and risk score were analyzed to assess the survival time at 1-, 3-, and 5-years for OSCC patients

**Fig. 8:**
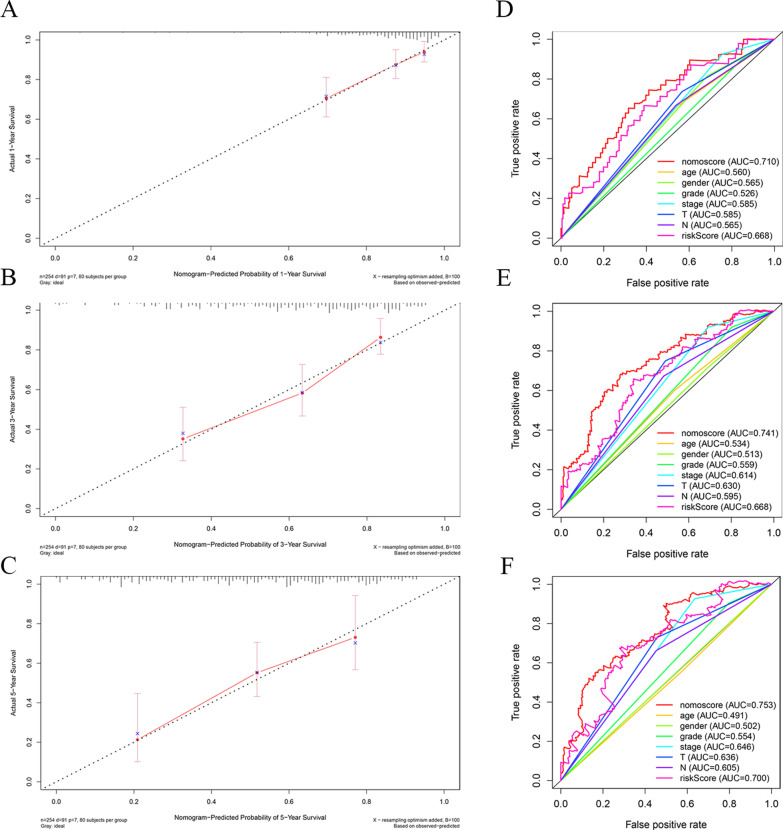
Evaluation of the nomogram. **A**–**C** Calibration curves of the nomogram for overall survival prediction at 1, 3, and 5 years. **D**–**F** The ROC curves for 1, 3 and 5 years overall survival of nomogram score, risk score and other clinical variables to evaluate the predictive ability of the nomogram

Based on the above predictors, we constructed a nomogram (Fig. 7). The calibration curves at 1, 3 and 5 years (Fig. 8a–c) approached 45°, which proves that the nomogram is accurate in predicting OSCC survival at 1, 3 and 5 years ROC curve analysis (Fig. 8d–f) was used to test the accuracy of the nomogram score, risk score, and clinical and pathological features at 1, 3 and 5 years. The results showed that relative to additional factors, like age, risk score, T, N stage and grade, the nomogram had higher accuracy in predicting prognosis at 1 year (AUC = 0.710), 3 years (AUC = 0.741) and 5 years(AUC = 0.753). According to the above results, our constructed nomogram well predicted OSCC survival.

### Enrichment analysis

**Fig. 9:**
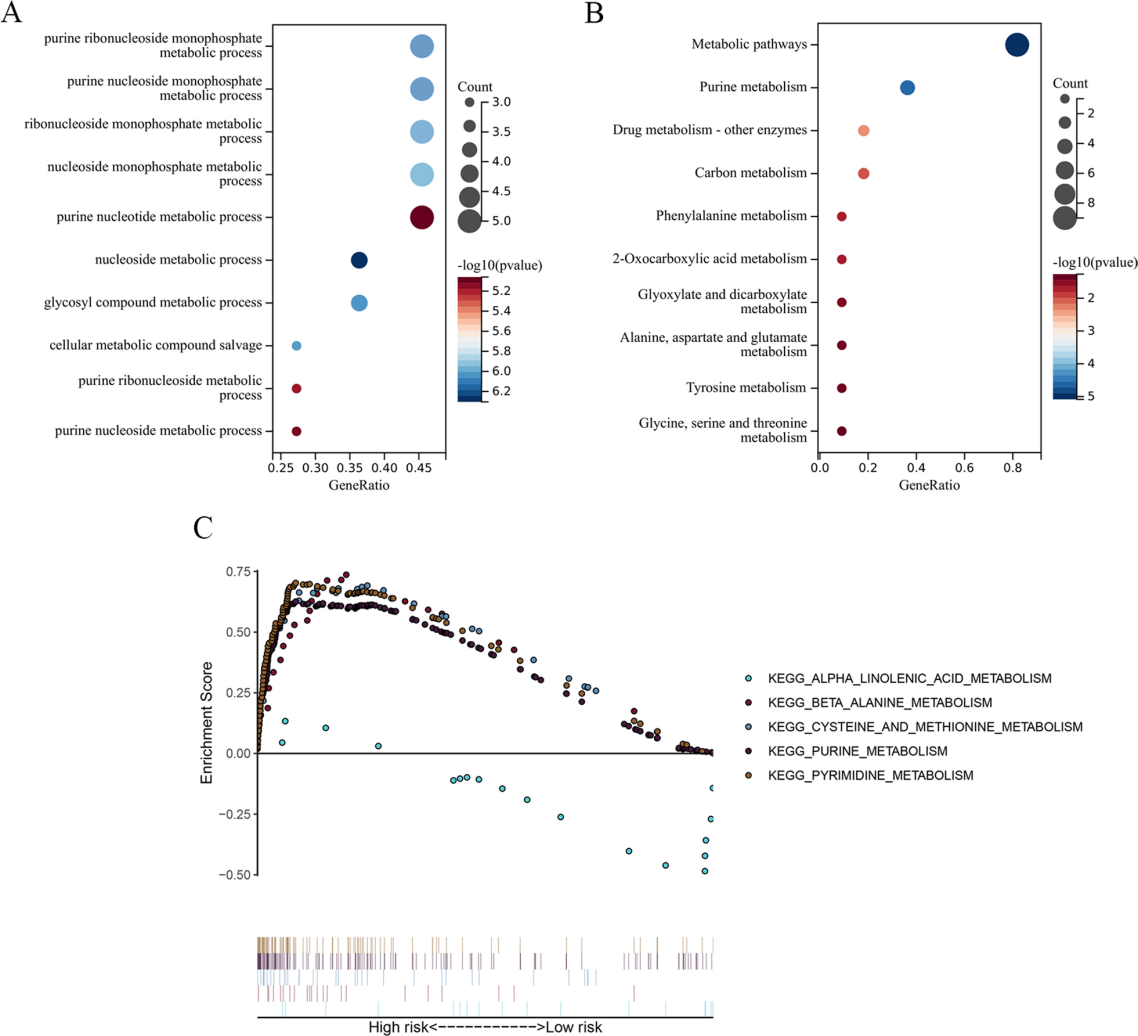
Gene functional enrichment analysis via GSEA of the eleven MRGs in the prognostic signature. **A** GO enrichment analysis of the eleven prognostic MRGs. **B** KEGG pathway enrichment analysis of the eleven prognostic MRGs. **C** Five representative KEGG pathways in high-risk patients and one representative KEGG pathways in low-risk patients

The function of 11 MRGs in OSCC biology was explored by GO as well as KEGG analysis. According to GO analysis results (Fig. 9a), those MRGs were mainly linked with purine ribonucleoside monophosphate metabolic process, purine nucleoside monophosphate metabolic process, nucleoside metabolic process, glycosyl compound metabolic process and so on. Upon KEGG analysis (Fig. 9b), MRGs were mostly associated with metabolic pathways, purine metabolism, drug metabolism-other enzymes, carbon metabolism, phenylalanine metabolism and so on. Next, we used GSEA software (Fig. 9c) to select five pathways markedly associated with high-risk patients, such as "β-Alanine metabolism", "cysteine and methionine metabolism", "purine metabolism", and "pyrimidine metabolism". The low-risk group showed α-linolenic acid metabolic enrichment. The above results illustrate the significantly different biological processes in low- versus high-risk patients.

### Association between risk signature and immune cell

**Fig. 10:**
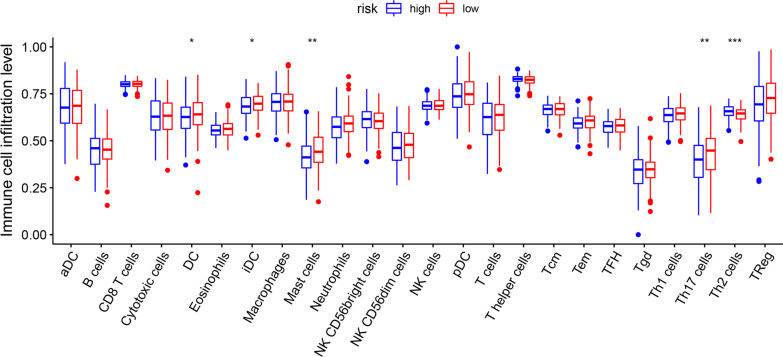
Immune infiltration cell score in high-risk and low-risk population

As shown in Fig. 10, dendritic cells (DC), immature dendritic Cells (iDC), mast cells, and T helper cell 17 (Th17) were more enriched in low-risk group (*P* < 0.05). T helper cell 2 (Th 2) were more enriched in high-risk group (*P* < 0.05). DC are the most potent antigen precursor cells in the immune system. DC-mediated cross-initiation of tumor-specific T cells plays a crucial role in initiating and maintaining anti-tumor immunity. Their presence in tumors tends to induce t-cell responses that slow cancer progression and is associated with improved patient survival [30]. Mast cells also play a multifaceted role in the tumor microenvironment by regulating multiple tumor biological events such as cell proliferation and survival, angiogenesis, invasion and metastasis [31]. These findings suggest that immune function is more active in low-risk group. Low-risk patients tend to have a better prognosis.

### A single-cell transcriptome atlas in OSCC

**Fig. 11:**
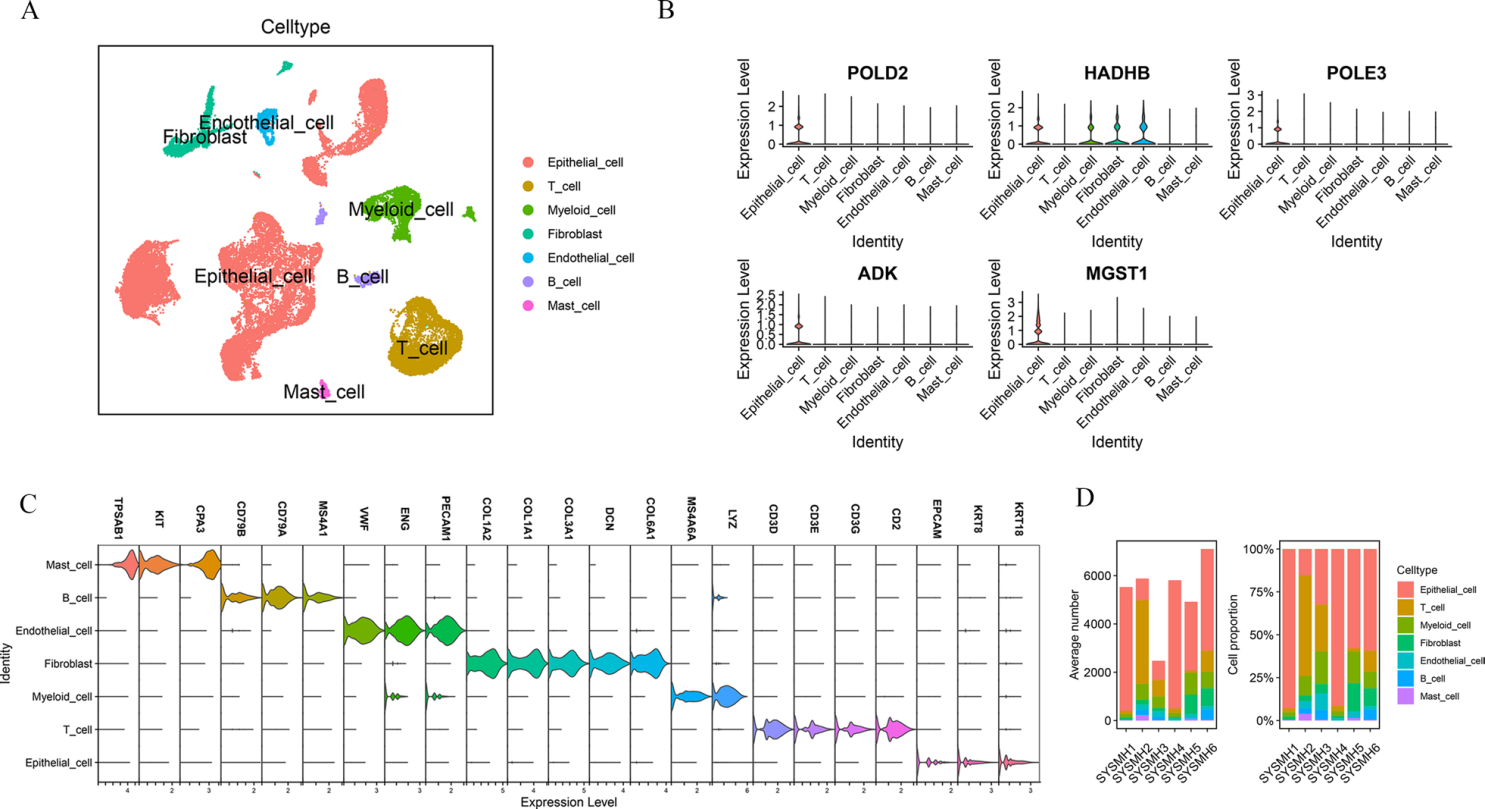
Cellular atlas of gastric tumours and non- tumour OSCC tissues. **A** UMAP plots showing cell types for the 31,719 cells. **B** The expression of five MRGs in OSCC cells **C** Violin plots showing the smoothed expression distribution of marker genes in seven cell types. **D** The proportion of each cell type in six samples

31,719 single cells were clustered into seven major cell types through marker genes: epithelial cells (marked with *EpCAM*, *KRT18* and *KRT8*); T cells (marked with *CD2*, *CD3G*, *CD3D* and *CD3E*); myeloid cell (marked with *LYZ*, *MS4A6*, *PECAM1*, *ENG*); fibroblasts (marked with *COL6A1*, *DCN*, *COL3A1*, *COL1A1* and *COL1A2*); endotheliocyte cell (marked with *PECAM1*, *ENG* and *VWF*); B cell (marked with *MS4A1*, *CD79A* and *CD79B*); mast cell (marked with *CPA3*, *KIT* and *TPSAB1*) (Fig. 11a and c). Then, we explored the expression of prognosis-related MRGs in various cells of OSCC (Fig. 11b). We found that five genes are basically expressed in epithelial cells. In other words, as epithelial-derived tumors, the abnormal metabolic patterns in OSCC tumor tissues may be limited to tumor cells. The other six MRGs that make up the prognostic model are not expressed in tumor microenvironment and tumor cells. The reason may be that single cell RNA-seq data usually contain many missing values caused by the failure of original RNA amplification. The proportion of each cell lineage varies greatly among different samples (Fig. 11d).

## Disscussion

Oral squamous cell carcinoma originates from oral keratinocytes. It has an insidious onset, making diagnosis difficult, and exhibits rapid development. Due to these characteristics, it is often not detected at an early stage; and the diagnosis of OSCC is usually made at the advanced stage, and the diagnosed patients usually develop distant metastasis upon diagnosis [32, 33]. Thus, accurate prognostic prediction is highly valuable, as it can aid in selecting the most suitable treatment for patients with OSCC, thereby improving their survival [34]. Therefore, it is significant to identify prognosis-related molecular markers comprehensively reflecting tumor biological features, so as to prevent and treat OSCC. With the development of bioinformatics, several large cancer databases, such as TCGA and GEO, have provided researchers access to large-scale gene expression data and corresponding clinical information [35]. Therefore, scholars can explore and develop new, more accurate prognostic models from the perspective of tumor cell biological behavior to optimize the treatment strategy of OSCC. An OSCC prognostic model constructed by using ferroptosis- and autophagy-related genes has been established to investigate the ability in predicting OSCC prognosis and the possibility of targeted treatment [36–38]. Many articles suggest the involvement of metabolism in OSCC genesis and progression, but little research regarding MRGs’effect on prognosis prediction of OSCC is available [39–41].

To analyze MRGs associated with OSCC survival, this work selected 12 prognostic MRGs from TCGA-OSCC cohort through univariate Cox and differential analyses. A clinical prognostic model of OSCC based on 11 MRGs was established by Lasso-Cox analysis, including 11 genes (*SHMT2*, *HPRT1*, *POLD2*, *HADHB*, *POLE3*, *ADK*, *GOT1*, *ATIC*, *MGST1*, *ADA* and *GNPDA1*).

Among these 11 genes, SHMT2, encoded by *SHMT2*, is an important enzyme related to carbon metabolism in OSCC, as it catalyzes the conversion of serine to glycine [42]. *SHMT2* level is discovered to be up-regulated in OSCC tissues, which has been linked to a poor prognosis; the increased expression predicts the worse the pathological state of the tumor [43]. In addition, the silencing of *SHMT2* in OSCC cells can affect cell cycle regulatory factors and induce cell G1 phase arrest, resulting in decreased cancer cell growth as well as inhibited cancer proliferation in vivo [44]. *SHMT2* is also highly expressed in thyroid cancer, bladder cancer and intrahepatic cholangiocarcinoma, and is closely related to the poor prognosis of these three cancers [45, 46]. *HPRT1* participates in regulating cell cycle mainly by regulating the production of purine and inosine in the remedial synthesis pathway [47]. The overexpression of *HPRT1* predicts the dismal survival of OSCC and enhances the resistance to cisplatin by promoting the MMP1/PI3K/AKT signaling pathway [48]. In addition, *HPRT1* overexpression dramatically decrease immunocyte activities, thus promoting the formation of an immunosuppressive tumor microenvironment [49]. *ADA* and *ADK* are mainly involved in adenosine metabolism. In the process of adenosine metabolism, *ADA* deaminates adenosine to yield inosine, and *ADK* phosphorylates adenosine to yield adenosine monophosphate [50, 51]. As indicated in this work, the levels of saliva ADA and serum ADA in OSCC cases markedly increased compared with healthy subjects, and the level of serum ADA increased with increasing histopathological grade [52, 53]. Some studies have confirmed that *ADA* is down-regulated in lymphocytes of advanced lung cancer [54]. *GOT1* is mainly involved in amino acid metabolism and the urea and tricarboxylic acid cycle [55, 56]. Another study confirmed that, in OSCC, *GOT1* is related to tumor invasion as well as shorter survival [57]. In addition, *GOT1* is also closely related to esophageal squamous cell carcinoma [58] and can be used as a biomarker of prostate cancer [59]. However, the relationship among *POLD2*, *HADHB*, *POLE3*, *ATIC*, *MGST1*, *GNPDA1* and OSCC remains unclear. *POLD2* and *POLE3* are necessary for DNA replication [60]. The expression of *PLOD2* has been discovered to be increased in patients with bladder cancer and ovarian cancer, which predicts dismal patient survival [61, 62]. *HADHB* is related to fatty acid metabolism and has been reported to be elevated in renal clear cell carcinoma and colorectal cancer [63, 64]. The expression of *ATIC* is abnormally upregulated in hepatocellular carcinoma patients, which has a potential tumor-promoting effect [65]. Overexpression of *MGST1* in tumor tissue can inhibit tumor cell apoptosis by inhibiting apoptosis-related signaling [66]. *GNPDA1* is mainly related to glycolysis and amino acid metabolism; studies have shown that *GNPDA1* is overexpressed in hepatocellular carcinoma [67]. It is speculated that the above genes could become prognostic markers of OSCC.

According to the results of these studies, we hypothesized that our identified gene signature composed of 11 MRGs could accurately predict OSCC prognosis. According to KM curve for training cohort, high-risk patients had markedly decreased OS compared with low-risk patients. The AUC of ROC curve for the risk score were 0.63, 0.70, 0.76 at 1, 3, and 5 years. A prediction models constructed by four autophagy-related genes perform well in predicting the overall survival of OSCC. The AUC values of these four genes was 0.65 [68]. Another study build a prognostic model for OSCC using 7 genes related to tumor mutational burden. From time-dependent ROC analysis, the AUC of 1, 3, and 5 years survivals of patients in the TCGA database were 0.67, 0.67 and 0.64 [69]. It indicated that this score predicted OSCC survival and that our as-constructed model performed well in predicting OSCC prognostic outcome. According to univariate as well as multivariate Cox regression on clinical features and risk scores of the patients, risk scores was the factor independently predicting prognosis. The 11 MRGs model performed well in prognosis prediction compared with additional clinicopathological characteristics alone. The above results were verified in the verification set, illustrating that our model has good universality and reliability. Finally, we established a nomogram by combining the risk model and the characteristics of clinical cases. The ROC curve and calibration curve both showed that our nomogram performed well in predicting OSCC prognosis. These findings suggest that combining risk score and additional clinical factors contributes to the accurate prediction of patient survival. As suggested by GSEA, the genes related to the regulation of metabolism were more highly associated with high-risk patients, which indicated the potent impact and regulation of MRGs on high-risk patients compared with low-risk counterparts. We also evaluated the relationship between immune infiltrating cells and risk scores, and the results suggest that the prognosis of the low-risk group seems to be better than that of the high-risk group. In addition, the results of single-cell sequencing and cell type identification show that the genes that make up OSCC prognosis model are limited to tumor cells rather than other cells. Collectively, our as-constructed 11 MRGs-based prognosis model may be adopted for predicting OSCC survival and providing more individualized treatment to OSCC patients.

MRGs can predict the prognosis of OSCC to some extent and are helpful for guiding patient treatment decision-making as well as follow-up visits. However, our study has some limitations. The data used in this study were acquired from public databases, so we cannot guarantee the integrity of patient data. In addition, due to the retrospective design of the study, there are potential biases related to imbalanced clinical characteristics. More experimental research and prospective works should be conducted for verifying MRGs’ prognosis prediction ability in OSCC.

## Conclusion

In this study, we screened 11 MRGs related to the prognosis of OSCC through a variety of bioinformatics methods. Based on these 11 MRGs, a nomogram combining age, gender, staging and risk score was established. This nomogram can help clinicians more accurately determine the prognosis of OSCC and provide more individualized treatment to these patients.

## Data Availability

The datasets used and/or analysed during the current study are available from NCBI Gene Expression Omnibus (GEO: GSE41613) https://www.ncbi.nlm.nih.gov/geo/ and the cancer genome database (TCGA: OSCC) https://portal.gdc.cancer.gov/.

## Abbreviations

OC: Oral cancer
OSCC: Oral squamous cell carcinoma
AJCC: American Joint Commission on Cancer
MRGs: Metabolism-related genes
TCGA: The Cancer Genome Atlas
GEO: Gene Expression Omnibus
OS: Overall survival
HRs: Hazard ratios
CIs: Intervals
KM: Kaplan–Meier
ROC: Receiver operating characteristic
AUC: Area under the curve
GO: Gene Ontology
KEGG: Kyoto Gene and Genomic Encyclopedia
